# The clinical translation of blood biomarkers for Alzheimer's disease: Timing and challenges

**DOI:** 10.1002/alz.70478

**Published:** 2025-08-05

**Authors:** Shan Huang, Jun Wang, Tengfei Guo, Yan‐Jiang Wang

**Affiliations:** ^1^ Department of Neurology and Center for Clinical Neuroscience Daping Hospital Army Medical University Chongqing China; ^2^ Chongqing Key Laboratory of Ageing and Brain Diseases Chongqing China; ^3^ Institute of Neurological and Psychiatric Disorders, Shenzhen Bay Laboratory Shenzhen Shenzhen China; ^4^ Chongqing Institute for Brain and Intelligence Guangyang Bay Laboratory Chongqing China; ^5^ State Key Laboratory of Trauma and Chemical Poisoning Chongqing China; ^6^ Key Laboratory of Geriatric Cardiovascular and Cerebrovascular Disease Research Ministry of Education of China Chongqing China

Dear Editor,

The clinical translation of blood biomarkers for Alzheimer's disease (AD) is both urgent and timely, though challenges remain. We appreciated Dr. Cui and Dr. Lu for their insightful comments on our previous study, which demonstrated that the Lumipulse G plasma p‐tau217 and p‐tau217/Aβ42 effectively identify abnormal amyloid‐beta (Aβ) and tau positron emission tomography (PET) status across clinical and community cohorts. They highlighted the urgency of clinically translating plasma p‐tau217 over further efficacy validation. Below, we address each of their subpoints in turn.

## 1 URGENCY AND CURRENT PROGRESS

### 1.1 The urgent need for AD diagnosis and screening: Advancing clinical practice and validation in parallel

The aging population has significantly increased the prevalence of Alzheimer's disease (AD), imposing a heavy societal burden.^1^ The recent approval of monoclonal antibody therapies targeting Aβ underscores the urgent need for cost‐effective, scalable tools for early diagnosis.^2^ Blood biomarkers, such as p‐tau217 and p‐tau217/Aβ42, have demonstrated diagnostic performance comparable to the United States Food and Drug Administration (FDA)‐approved cerebrospinal fluid (CSF) biomarkers, offering a less invasive and scalable alternative.^3^ We advocate for AD blood biomarkers to advance clinical practice and validation in parallel.

### 1.2 High‐sensitivity and automated analytical platforms drive clinical translation of AD blood biomarkers

Technological advancements in ultra‐sensitive platforms have propelled AD biomarker research, overcoming limitations of classical methods like enxye‐linked immunosorbent assay (ELISA). Current platforms such as chemiluminescence immunoassay (CLIA), electrochemiluminescence (ECLIA), immunoprecipitation‐liquid chromatography‐tandem mass spectrometry (IP‐LC‐MS/MS), and single molecule array (SIMOA) demonstrate high diagnostic accuracy for blood p‐tau217 and p‐tau217/Aβ42.^4^, ^5^ Fujirebio Lumipulse G, the first FDA‐approved blood‐based AD diagnostic assay, offers high sensitivity, automation, and rapid detection, though challenges include reagent variability and limited in‐house development. Roche Elecsys also provides high sensitivity and automation, with electrochemiluminescence technology reducing signal interference and sample variability, but it is hindered by high costs and large sample requirements. IP‐LC‐MS/MS minimizes antibody dependency and variability but remains complex, expensive, and time‐intensive. SIMOA enables ultra‐sensitive detection for low‐concentration biomarkers but requires further validation for stability and cost efficiency.

### 1.3 A two‐step diagnostic workflow

A two‐cutoff approach with an intermediate gray zone improves diagnostic flexibility and accuracy. When the gray zone is controlled within 20%, plasma p‐tau217/Aβ42 can still achieve 95% diagnostic accuracy.^3^ A proposed two‐step workflow involves: Initial screening: results are categorized as positive, negative, or intermediate based on thresholds; Follow‐up testing: intermediate results undergo confirmatory positron emission tomography (PET) imaging or cerebrospinal fluid (CSF) testing. Replicate testing and multi‐biomarker panels further reduce variability, while combining biomarker results with comprehensive clinical assessment enhances diagnostic precision, especially for rare AD subtypes or overlapping comorbidities.^6^

## 2 CHALLENGES AND FUTURE DIRECTIONS

### 2.1 Limited specificity of p‐tau217

P‐tau217 is not entirely AD‐specific. For conditions with elevated p‐tau217 levels (e.g., Lewy body dementia) or AD subtypes without significant p‐tau217 elevation, combining multiple biomarkers can improve diagnostic accuracy.^6^, ^7^ Future research should also focus on developing blood‐based assays for Aβ, which is highly specific to AD but currently limited by peripheral metabolism effects and small fold changes in blood Aβ42/40 ratios.^8^ Advancements may include exploring AD‐specific Aβ subtypes, such as pyroglutamylated Aβ, and optimizing sampling from sites less affected by peripheral metabolism, such as the internal jugular vein, to improve the clinical utility of blood‐based Aβ testing.

### 2.2 Standardization of AD blood biomarkers

The inherent characteristics of AD biomarkers make them highly vulnerable to variations in the preprocessing and testing process. For instance, Aβ42 is prone to adhesion, while p‐tau is temperature‐sensitive. Standardized sample preprocessing workflows, testing protocols, and rigorous quality control systems for AD blood biomarkers are essential prerequisites for clinical application. Guidelines from organizations such as the International Alzheimer's Association and the Standardization of Alzheimer's Blood Biomarkers working group provide a foundation for standardized sample preprocessing.^9^ Automated platforms have significantly improved the standardization of testing workflows, with platform‐specific pre‐loading procedures. Certified reference materials are crucial for ensuring cross‐platform result comparability, and regional or ethnic variations in biomarker levels must also be considered when defining thresholds or interpreting results. Rigorous quality control (QC) involves using in‐house QC samples with varying biomarker concentrations, prepared with the same specimen type as test samples, alongside kit‐provided controls to detect system or operator variability. For consistent performance monitoring, the same QC batch should be used over time, and bridging studies are necessary when transitioning to a new batch to maintain data comparability.^10^

## 3 CONCLUSION

Accelerating the clinical translation of AD blood biomarkers is essential at this pivotal stage. With ongoing advancements in detection technologies, artificial intelligence (AI)‐driven multimodal data integration, and strengthened international multicenter collaborations, blood biomarkers have the potential to revolutionize the diagnostic and therapeutic paradigm of AD.

## CONFLICT OF INTEREST STATEMENT

The authors declare no competing interests. Author disclosures are available in the supporting information.

## Supporting information



Supporting Information
